# Antiviral and Antioxidant Properties of Echinochrome A

**DOI:** 10.3390/md16120509

**Published:** 2018-12-15

**Authors:** Sergey A. Fedoreyev, Natalia V. Krylova, Natalia P. Mishchenko, Elena A. Vasileva, Evgeny A. Pislyagin, Olga V. Iunikhina, Vyacheslav F. Lavrov, Oksana A. Svitich, Linna K. Ebralidze, Galina N. Leonova

**Affiliations:** 1 G.B. Elyakov Pacific Institute of Bioorganic Chemistry, FEB RAS, Vladivostok 690022, Russia; mischenkonp@mail.ru (N.P.M.); vasilieva_el_an@mail.ru (E.A.V.); pislyagin@hotmail.com (E.A.P.); 2 G.P. Somov Institute of Epidemiology and Microbiology, FEB RAS, Vladivostok 690087, Russia; krylovanatalya@gmail.com (N.V.K.); olga_iun@inbox.ru (O.V.I.); galinaleon41@gmail.com (G.N.L.); 3 I.I. Mechnikov Research Institute of Vaccines and Sera, Moscow 105064, Russia; v.f.lavrov@inbox.ru (V.F.L.); svitichoa@yandex.ru (O.A.S.); lina.lidze@gmail.com (L.K.E.)

**Keywords:** echinochrome A, composition of antioxidants, antioxidant activity, antiviral activity

## Abstract

The aim of this study was to examine the in vitro antioxidant and antiviral activities of echinochrome A and echinochrome-based antioxidant composition against tick-borne encephalitis virus (TBEV) and herpes simplex virus type 1 (HSV-1). The antioxidant composition, which is a mixture of echinochrome A, ascorbic acid, and α-tocopherol (5:5:1), showed higher antioxidant and antiviral effects than echinochrome A. We suppose that echinochrome A and its composition can both directly affect virus particles and indirectly enhance antioxidant defense mechanisms in the hosting cell. The obtained results allow considering the echinochrome A and the composition of antioxidants on its basis as the promising agents with the both antioxidant and antiviral activities.

## 1. Introduction

Oxidative stress, arising through production of free radicals including reactive oxygen species (ROS), is usually defined as a disturbance in the balance between the level of ROS and antioxidant defenses [1]. Viral infections, along with other numerous human diseases, are accompanied by oxidative stress, which plays an important role in their pathogenesis [2,3]. Oxidative processes promote virus replication in infected cells, decrease cell proliferation, and induce cell apoptosis [4]. Intensification of the processes of free radical lipid oxidation and the sharp suppression of the antioxidant and antiradical protection system of the body are observed in patients with neurotropic virus infections such as tick-borne encephalitis [5] and herpes simplex [6,7]. Central nervous system tissues are especially sensitive to lipid peroxidation due to their high lipid content [8]. The lipid peroxides resulting from the ROS-induced peroxidation of membrane phospholipids, such as malondialdehyde, can transverse the circulation and cell membranes, with the resultant dysfunction of vital cellular processes such as membrane transport and mitochondrial respiration [9].

Antioxidants with different mechanisms of action are used to prevent or treat various diseases that are associated with oxidative stress and possess therapeutic effects in many cases [10,11,12]. Since the most important aspect of the treatment of viral diseases is the suppression of viral replication followed by cell survival, the search for drugs that have antiviral properties among antioxidants is promising. There are many examples showing that natural antioxidants such as vitamins C and E (ascorbic acid and α-tocopherol, respectively), curcumin, various polyphenols, and others are promising agents for antiviral therapy, since they decrease ROS levels in infected cells, the expression of pro-apoptotic signaling molecules, and modulate the cellular levels of stress-related proteins such as c-Jun N-terminal kinases (JNK), phospho-p38 mitogen-activated protein kinase (MAPK), extracellular signal-regulated kinases (ERK-1/2), and transcription factor NF-kB [13,14,15,16,17,18].

A well-known natural antioxidant echinochrome A (naphthoquinonoid pigment of sea urchins) is the active substance of the Russian drug Histochrome^®^, which is used in cardiology for the treatment of ischemic heart disease and myocardial infarction, and in ophthalmology for the treatment of degenerative diseases of the retina and cornea, macular degeneration, primary open-angle glaucoma, and others [19,20].

The aim of this research was to study the in vitro antioxidant and antiviral activities of echinochrome A (Ech) and the compositions based on Ech, including also other antioxidants, against RNA-containing tick-borne encephalitis virus (TBEV) and DNA-containing herpes simplex virus type 1 (HSV-1).

This paper was prepared for printing on the basis of materials presented as a lecture on the Third International Symposium on Life Science, Vladivostok, Russia, September 2018.

## 2. Results

### 2.1. Antioxidant Activity of Ech Formulations Alone or Combined with Other Antioxidants

We have compared antioxidant properties of Ech, α-tocopherol (Toc), and ascorbic acid (Asc), as well as their combinations, using the model of linetol peroxidation. The procedure that we applied relates to simple gravimetric methods via the measurement of weight increases following oxygen fixation on fatty acids [21]. Action of the studied substances on linetol was characterized as the induction time of the lipid auto-oxidation reaction (Δτ, h-difference between times necessary for linetol oxidation in the presence and absence of an antioxidant). The determination of antioxidant activities made it possible not only to compare the antioxidant activities of the studied substances with each other, but also to find the optimal ratio of antioxidants in the most active compositions. It was established that Toc was the most effective antioxidant in this experiment (Δτ 125 h) (Table 1). Ech was some less effective (Δτ 100 h), while Asc showed no antioxidant effect on this model. The low efficiency of Asc may be explained by its high susceptibility to auto-oxidation in linetol solution. It is known that in experiments in vitro, Asc lacks antioxidant activity in the absence of Toc. This observation was confirmed by our experiments (Δτ of the mixture Asc + Toc (2:1) was 195 h, which is more than effect of Toc itself). A mixture of all three antioxidants (Ech + Asc + Toc) demonstrated a stronger effect on a model of linetol auto-oxidation as a result of the synergy of these compounds (Δτ 223 h) (Table 1). We calculated the effect of synergism (in %) according to Kancheva et al. by the formulas for binary and ternary mixtures of antioxidants [22].

### 2.2. Antioxidant Activity of the Formulations Against LPS-Induced ROS Formation in Vero Cells

**Figure 1:**
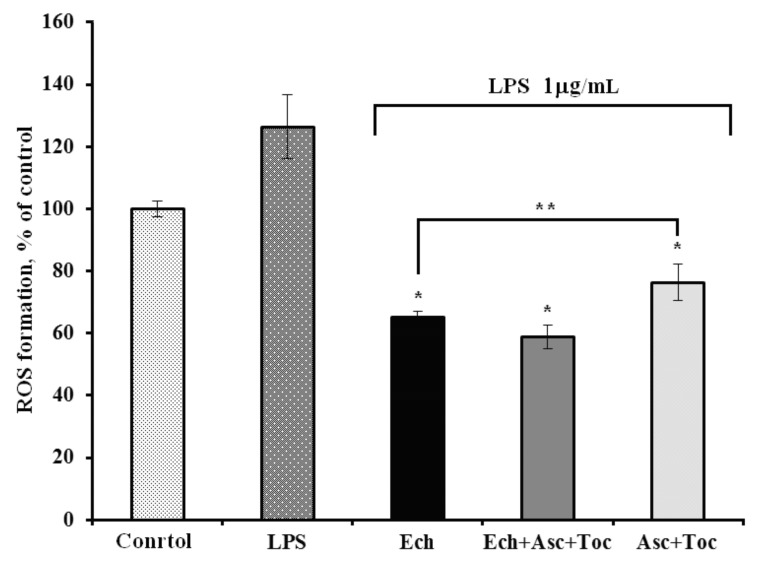
Influence of Ech and studied formulations on the lipopolysaccharide (LPS)-induced reactive oxygen species (ROS) formations in Vero cells. The formulations were tested at a concentration of five μg/mL. * *p* < 0.05; ** statistically significant differences between Asc + Toc and Ech (*p* ≤ 0.05).

To determine whether Ech alone or combined with other antioxidants is able to decrease intracellular ROS level in Vero cells, we used the model of *E. coli* lipopolysaccharide (LPS)-induced ROS formation. The ROS levels in Vero cells treated with LPS increased by 20% in comparison to control–untreated cells (Figure 1). Ech, Ech + Asc + Toc, and Asc + Toc decreased the ROS formation by 61%, 68%, and 50% in Vero cells, correspondingly, in comparison to LPS-treated cells.

Ech and its composition with Asc and Toc showed significant antioxidant effects on both experimental models, which makes them promising agents for further investigations on TBEV and HSV-1 replications accompanied by oxidative stress.

### 2.3. Cytotoxicity and Antiviral Activity of Formulations.

Cytotoxicity assay was carried out to determine the concentration range of formulations for the subsequent study of its antiviral activity in the non-toxic range for pig embryo kidney (PK) and Vero cells. Acyclovir and ribavirin were used as standard antivirals for HSV-1 and TBEV, respectively. Based on the obtained methylthiazolyltetrazolium bromide (MTT) assay results, 50% cytotoxic concentrations (CC_50_) against PK and Vero cells were determined for all of the studied formulations (Table 2). Further antiviral activity assay was performed at the concentrations of the formulations below 400 μg/mL.

The anti-TBEV and anti-HSV-1 activity of tested formulations were assessed using cytopathic effect (CPE) inhibition assay. PK and Vero cells infected with the 10-fold dilutions of corresponding virus were simultaneously treated with different concentrations of the formulations. It was found that the formulations inhibited virus-induced CPE in a dose-dependent manner, and values of the 50% inhibitory concentrations (IC_50_) and selective indices (SI) of the tested formulations for both viruses are presented in the Table 2. Ech and the Ech + Asc + Toc composition revealed moderate antiviral activities against TBEV and HSV-1 compared with Asc + Toc. Furthermore, based on IC_50_ and SI values, the Ech + Asc + Toc composition was more active toward TBEV and HSV-1 than Ech and Asc + Toc (*p* ≤ 0.05) (Table 2). The obtained data revealed that the presence of Asc and Toc in composition with Ech enhances antiviral activity of this formulation up to two times compared with Ech alone.

### 2.4. Time-of-Formulation-Addition Assay

**Figure 2:**
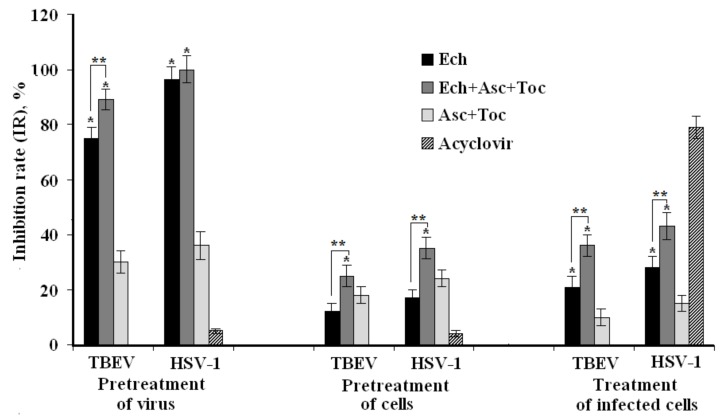
Antiviral action of the formulations on different stages of virus replication cycles. * Statistically significant differences between Asc + Toc and other formulation (*p* ≤ 0.05), ** statistically significant differences between antioxidant composition and Ech (*p* ≤ 0.05).

The inhibitory effects of tested formulations on different stages of TBEV and HSV-1 replication cycles were studied by time-of-addition experiments via MTT assay (Figure 2). Cells were pretreated with formulations before viral infection (pretreatment of cells), viruses were incubated with formulations before cell infection (pretreatment of virus), or infected cells were incubated with formulations after penetration of the virus into host cells (treatment of infected cells).

In the case of the pretreatment of viruses with the formulations (direct virucidal effect), Ech and the Ech + Asc + Toc composition considerably suppressed TBEV infection: inhibition rates (IR) were of 75 ± 4% and 89 ± 5%, respectively (*p* < 0.05). The corresponding pretreatment of HSV-1 by Ech and the Ech + Asc + Toc composition completely protected cells against this infection. However, only a minor effect on HSV-1 infection was detected when the virus was pretreated with acyclovir (Figure 2).

The treatment of PK and Vero cells with the tested formulations before infection (preventive effect) was much less effective. Ech, the Ech + Asc + Toc composition, and the Asc + Toc composition showed almost no preventive action against both virus infections. The same results were found when Vero cells were pretreated with acyclovir prior to infection.

When the formulations were added at an early stage of virus replication (one hour after infection), Ech and the Ech + Asc + Toc composition possessed moderate virus-inhibiting effects against TBEV infection with an IR of 21 ± 2% and 36 ± 3%, respectively, and against HSV-1 with an IR of 28 ± 3% and 43 ± 4%, respectively, compared to an inactive Asc + Toc formulation (~10%, *p* < 0.05). Meanwhile, acyclovir showed the highest antiviral activity, with an inhibition of the HSV-1 replication of 79 ± 4%.

## 3. Discussion

It was of interest that the Ech + Asc + Toc composition, which included three different antioxidants, demonstrated the most potent antioxidant action. Therefore, this composition showed a pronounced synergistic effect, which means that its antioxidant activity was much higher compared to each of the components added in the same amount to stabilize the lipid substrate due to the continuous regeneration of Toc from both the Ech and Asc. Moreover, the synergistic action of Ech in combination with other antioxidants was not so far reported. Ech and the Ech + Asc + Toc composition demonstrated high antioxidant activity on the model of LPS-induced ROS formation in Vero cells.

Since Ech and its composition with Asc and Toc showed significant antioxidant effects on both experimental models, and because of the ability of Ech to overcome the blood–brain barrier [23], further investigations of their effects on the replication cycles of neurotropic viruses such as TBEV and HSV-1 accompanied by oxidative stress were performed. Earlier combinations of antiviral agents with antioxidants have been used for the treatment of some other viral infections, for example, at influenza-associated complications [24]. In this study, we have shown the possibility of enhancing the antioxidant and antiviral effects of Ech due to combination with other antioxidants. We have found that the most effective method of application of Ech and the studied composition is the pretreatment of viruses with the formulations (virucidal action). The antioxidant composition Ech + Asc + Toc also demonstrated stronger antiviral activity against TBEV and HSV-1 compared to Ech. The inhibitory concentrations (IC_50_) of the composition were half that of Ech, while the selective indices (SI) were twice as large as those of Ech (Table 2). It should be noted that regardless of the method of exposure of viruses and cells to the formulations, the virus inhibition rates of the composition of antioxidants was higher than that by Ech itself (*p* < 0.05, Figure 1).

It was shown that the main mechanism of the in vitro action of Ech and the composition of antioxidants at the stages of the life cycles of TBEV and HSV-1 is a direct inactivation of virus particles (Figure 2). Many authors have suggested that the virucidal activity of polyphenols (Ech is considered a polyphenol as well) might be caused by direct action on the viral particles inhibiting the adsorption of the virus to the host cell receptors [25,26,27]. At the same time, Li et al. reported that polyphenols can cause irreversible damage or the reversible blocking of certain regions of the viral capsid protein [28]. We suppose that Ech and its composition with antioxidants can bind with some envelope virus proteins that are necessary for the adsorption of the virus to cells. Since many polyphenols exhibit antioxidant and antiviral properties, we can assume that the activity of Ech and its composition in relation to TBEV and HSV-1 can also be caused by interfering with the redox imbalance caused by these viruses [16]. Thus, Ech, either alone or in varying compositions, can both directly affect virus particles and indirectly enhance the antioxidant defense mechanisms in the hosting cell.

Thus, the ability of Ech and its compositions to inactivate HSV-1 and TBEV virus particles makes it useful as an antiviral agent in preventing de novo viral infection, and thereby could help control viral spread and limit recurrent infections.

Our data on the antioxidant and antiviral activities of Ech—which earlier have been applied to the treatment of cardiovascular and eye diseases—and antioxidant composition based on Ech indicate the necessity of further studies of these formulations for the development of promising antiviral drugs.

## 4. Materials and Methods

### 4.1. Viruses and Cell Cultures

The RNA-containing tick-borne encephalitis virus (TBEV) strain Dal’negorsk that was isolated in 1973 from the brain of a patient with a fatal outcome of TBE, and characterized as a Far Eastern subtype, was used (Gene Bank Whole Genome Sequence Number: FJ402886) [29]. The DNA-containing herpes virus (HSV-1, strain VR3) was obtained from the National Collection of US Viruses (Rockville, MD, USA).

TBEV was grown on the pig embryo kidney (PK) cells using medium 199 supplemented with 10% fetal bovine serum (FBS) and 100 U/mL gentamicin. HSV-1 was grown in African green monkey kidney (Vero) cells using Dulbecco’s Modified Eagle’s Medium (DMEM) supplemented with 10% FBS, gentamicin, and glutamine.

Viral titers were determined by cytopathic effect (CPE) assay and expressed as the 50% tissue culture infectious dose (TCID_50_/mL). The TBEV titer was 10^8.8^ TCID_50_/mL, and the titer of HSV-1 was 10^8.25^ TCID_50_/mL.

### 4.2. Studied Formulations

Echinochrome A (Ech, 2,3,5,7,8-pentahydroxy-6-ethyl-1,4-naphthoquinone) 98.0%, pharmaceutical, state registration number РN002362/01-2003, G.B. Elyakov Pacific Institute of Bioorganic Chemistry FEB RAS, Russia.

Ascorbic acid (Asc) 99.8%, pharmaceutical, AppliChem, Germany.
α-Tocopherol (Toc) ≥96%, pharmaceutical, Carl Roth, Germany.
The composition of antioxidants Ech + Asc + Toc at the weight ratio of 5:5:1.
The composition containing Asc and Toc at the weight ratio of 5:1.
Ribavirin^®^, pharmaceutical, Vertex, Russia.
Acyclovir^®^, pharmaceutical, Belmedpreparation, Republic of Belarus.

The tested formulations were dissolved in dimethylsulfoxide (DMSO, Sigma, Saint-Louis, MO, USA) and stored at −20 °C. The stock solutions (10 mg/mL) of formulations were diluted with a suitable cell culture medium so that the final concentration of DMSO was 0.5%.

### 4.3. Determination of Antioxidant Activity of the Formulations

Antioxidant activity of the formulations was determined on a model of linetol peroxidation containing a complex mixture of ethyl esters of polyunsaturated fatty acids (oleic, linoleic, and linolenic) of linseed oil at 37 °C [30]. Stock solutions of Ech, Asc, and Toc were prepared at a concentration of 10 mg/mL in ethanol. Two-component and three-component antioxidant compositions were obtained by mixing the volumes of stock solutions in the indicated proportions. First, 10 μL of each solution and 300 μL of linetol were placed in a glass vial. The reaction vessels were placed in an incubator (37 °C). The concentration of antioxidant in linetol in all cases was 0.05 mg/mL, or 0.005%. The mass of the reaction mixtures pre-cooled to room temperature was measured twice a day (accuracy 0.0005 g). When the mass of increased by 10 mg, the reaction was stopped. All of the experiments were repeated three times. The period of linetol oxidation inhibition (Δτ) was calculated as the difference between times necessary for the weight of linetol to increase by 10 mg in experiments with and without the addition of antioxidants using the formula Δτ = τ − τ_0_, where τ is the time of linetol oxidation initiation in the presence of an antioxidant (h); and τ_0_ is the time of linetol oxidation initiation without the addition of an antioxidant (h).

### 4.4. Antioxidant Activity of the Formulations Against LPS-Induced ROS Formation in Vero Cells

The Vero cells that were grown on 96-well plates (1 × 10^4^ cells/well) were washed from the growth medium and treated with 100 µL/well of the tested compounds (five μg/mL) and 10 µL/well LPS from *E. coli* serotype 055:В5 (Sigma, 1.0 μg/mL), which were both dissolved in PBS and cultured at 37 °C in a CO_2_-incubator for one hour. For the ROS levels measurement, 20 μL of 2,7-dichlorodihydrofluorescein diacetate (DCF-DA, Sigma, final concentration 10 μM) solution was added to each well, and the plates were incubated for 30 min at 37 °C. The intensity of DCF-DA fluorescence was measured at λ_ex_ 485 n/λ_em_ 518 nm using the plate reader PHERAstar FS (BMG Labtech, Offenburg, Germany) [31].

### 4.5. Cytotoxicity Assay of the Formulations

The cytotoxicity of the tested formulations was estimated by MTT assay in PK and Vero cell lines [32,33]. A monolayer of cells (1 × 10^4^ cells/well) grown in 96-well plates was treated with different concentrations of tested formulations (from 0 to 2000 µg/mL) and cultured at 37 °C in a CO_2_-incubator for six days; untreated cells were used as controls. Then 20 μL of MTT solution (5 mg/mL) (methylthiazolyltetrazolium bromide, Sigma, Saint-Louis, MO, USA) was added in each well followed by incubation at 37 °C for one hour. The MTT solution was removed, and 150 µL/well of isopropanol was added. Optical density (OD) was measured at 540 nm using an ELISA microplate reader (Labsystems Multiskan RC, Vantaa, Finland) with a reference absorbance at 620 nm. The viability of the cells was calculated as (ODt)/(ODc) × 100%, where ODt and ODc correspond to the absorbance of treated and control cells, respectively. Cytotoxicity was expressed as 50% cytotoxic concentration (CC_50_) of the tested formulation that reduced the viability of treated cells by 50% compared with control cells. Experiments were performed in triplicate and repeated three times.

### 4.6. Antiviral Activity ASSAY of Formulations

The antiviral activity of formulations against TBEV and HSV-1 was evaluated using cytopathic effect (CPE) inhibition assay in PK and Vero cells, respectively. The overnight monolayer of cells grown on 96-well plates (1 × 10^4^ cells/well) was infected with 100 µl/well of serial dilutions of virus suspension (10^−1^–10^−8^) and simultaneously treated by formulations (100 μL/well in triplicate) of different concentrations (from 0 to 400 µg/mL) for one hour at 37 °C. After virus absorption, the virus–formulations mixture was removed; the cells were washed, and a maintenance medium with 1% FBS was added. The plates were kept at 37 °C in CO_2_-incubator for six days for TBEV or for three days for HSV-1 until CPE appeared. The antiviral activity was determined by the difference of the viral titers between treated infected cells and untreated infected cells and expressed as the inhibition rate (IR, %), using the formula [27]: IR = (1 − *T*/*C*) × 100, where *T* is the antilog of the formulations-treated viral titers and *C* is the antilog of the control (without formulations) viral titers. The concentration of formulation that reduced the virus-induced CPE by 50% was determined as the 50% inhibitory concentration (IC_50_). The selectivity index (SI) was calculated as the ratio of CC_50_ to IC_50_. Experiments were repeated three times.

### 4.7. Time-of-Formulation-Addition assay

PK and Vero cells were grown in 96-well plates (1 × 10^4^ cells/well). An infectious dose of both TBEV and HSV-1 was of 100 TCID_50_/mL, tested formulations were used at a concentration of 20 μg/mL, and acyclovir was used at a concentration of 10 μg/mL. The plates were kept at 37 °C in CO_2_-incubator for six days for TBEV or for three days for HSV-1 until 80–90% CPE was observed in viruses control compared with cells control.

- Pretreatment of cells with formulations. Monolayer of cells was pretreated with formulations in triplicate and incubated at 37 °C for one hour. Thereafter, the cells were washed and infected with virus at 37 °C for one hour. The cells were washed to remove unabsorbed virus and incubated with maintenance medium until CPE was observed.
- Pretreatment of virus with formulations. The virus was mixed with formulations at a ratio 1:1 (*v*/*v*), incubated for one hour at 37 °C, then applied to monolayer of cells in triplicate. After one hour adsorption at 37 °C, cells were washed, and maintenance medium was added, followed by incubation until CPE was observed.
- Treatment of infected cells. Monolayer of cells was infected with the virus at 37 °C for one hour, then washed, treated with tested formulations in triplicate, and incubated until CPE was appeared.

Antiviral activity of formulations was assessed by MTT test, and the viral inhibition rate (IR, %) was calculated according to the formula [34], IR = (ODtv − ODcv)/(ODcd − Odcv) × 100, where ODtv represents the OD of cells infected with virus and treated with the test formulation; ODcv corresponds to the OD of the untreated virus-infected cells, and ODcd is OD of control (untreated and noninfected) cells.

### 4.8. Statistical Analysis

CC_50_ and IC_50_ were calculated by regression analysis of the dose–response curve. Statistical processing of the data was performed using Statistica 10.0 software. The results are given as mean ± standard deviation (SD). The differences between parameters of control and experimental groups were estimated using the Wilcoxon test. Differences were considered significant at *p* ≤ 0.05.

## 5. Conclusions

We have shown that the both Ech, an active substance of the permitted to clinical application in Russia drugs, belonging the Histochrome series, and an antioxidant composition, containing Ech, Asc, and Toc (5:5:1), possess in vitro antiviral activity against RNA-containing tick-borne encephalitis virus and DNA-containing herpes simplex virus type 1. The studied composition of antioxidants exhibits more potent antioxidant and antiviral properties than Ech itself, thus demonstrating the synergistic effects of its components.

## Figures and Tables

**Table 1:** Antioxidant activity of the formulations on a model of linetol auto-oxidation. ^1^

Antioxidants and their Compositions	Δτ, h	The Efficiency of the Composition Compared to Ech
Ech	100 ± 5	-
Asc	24 ± 3	-
Toc	125 ± 7	-
Ech + Asc (1:1)	69 ± 4	No effect
Ech + Toc (1:1)	201 ± 8 *	Synergism 8%
Asc + Toc (2:1)	195 ± 7 *	Synergism 7%
Ech + Asc + Toc (5:5:1)	223 ± 10 **	Synergism 40%
Control-linetol	24 ± 2	

^1^ The concentration of Ech, Asc, Toc and their compositions in test medium was of 0.05 mg/mL. * Statistically significant differences between Ech and antioxidant compositions (*p* ≤ 0.05); ** statistically significant differences between three-component and two-component mixtures of antioxidants (*p* ≤ 0.05). Asc: ascorbic acid, Ech: echinochrome A, Toc: α-tocopherol.

**Table 2:** Cytotoxic and antiviral activities of formulations against tick-borne encephalitis virus (TBEV) and herpes simplex virus type 1 (HSV-1).

Formulation	TBEV			HSV-1		
	CC_50_ (µg/mL)	IC_50_ (µg/mL)	SI	CC_50_ (µg/mL)	IC_50_ (µg/mL)	SI
Ech + Asc + Toc (5:5:1)	57.9 ± 2.3 *	12.6 ± 1.5 **	4.8 ± 0.5 **	66.7 ± 3.2 *	11.2 ± 1.2 **	6.0 ± 0.6 **
Ech	54.4 ± 1.8 *	21.8 ± 2.6 *	2.5 ± 0.2 *	60.5 ± 3.1 *	18.8 ± 2.1 *	3.2 ± 0.3 *
Asc + Toc (5:1)	521.7 ± 5.3	1304 ± 145	0.4 ± 0.1	530.9 ± 9.4	885 ± 97	0.6 ± 0.1
Ribavirin	2010 ± 180	30.5 ± 4.6	66.0 ± 5.4			
Acyclovir				1470 ± 160	10.8 ± 1.2	133.6 ± 12.0

CC_50_-50% cytotoxic concentration of a formulation, IC_50_-50% virus-inhibiting concentration of a formulation, SI: selective index of the formulation. * Statistically significant differences between Asc + Toc and other formulation (*p* ≤ 0.05), ** statistically significant differences between antioxidant composition and Ech (*p* ≤ 0.05).
